# Alkali Attack on Anion Exchange Membranes with PVC Backing and Binder: II Prediction of Electrical and Mechanical Performances from Simple Optical Analyses

**DOI:** 10.3390/membranes8040133

**Published:** 2018-12-14

**Authors:** Shoichi Doi, Maki Kinoshita, Masahiro Yasukawa, Mitsuru Higa

**Affiliations:** 1 Graduate School of Sciences and Technology for Innovation, Yamaguchi University, 2-16-1 Tokiwadai, Ube, Yamaguchi 755-8611, Japan; myasu@yamaguchi-u.ac.jp; 2 Astom Corporation, 1-1 Mikagecho, Syunan, Yamaguchi 745-8648, Japan; s.doi@astom-corp.jp (S.D.); m.kinoshita@astom-corp.jp (M.K.); 3 Blue Energy Center for SGE Technology (BEST), 2-16-1 Tokiwadai, Ube, Yamaguchi 755-8611, Japan

**Keywords:** anion exchange membrane, alkaline cleaning, polyvinyl chloride, relative total VIS reflectance, X-ray fluorescence

## Abstract

Performance of anion exchange membranes (AEMs), including polyvinyl chloride (PVC) as backing and binder, decreases during a repetitive cleaning-in-place (CIP) treatment using alkali. In this study, we have systematically performed two optical analyses, relative total visible (VIS) reflectance and handheld X-ray fluorescence (XRF), for alkali-attacked commercially available AEM (Neosepta^®^ AMX, Tokyo, Japan) with different NaOH immersion conditions (0–1.0 M NaOH at 40–80 °C for 0–168 h). The VIS reflectance and XRF data were then compared with the electrical and mechanical performances (i.e., membrane resistance, proton rejection, amount of fixed-charge sites, and Young’s modulus) of the alkali-attacked AMXs. The result indicated that there are clear linear relationships between their performances and both VIS reflectance and XRF data especially at 40 °C, indicating both optical analyses have a good possibility as a quick diagnosis-in-place (DIP) to predict the resulting performance of the alkali-attacked AMXs. In addition, we also found a clear linear relationship between VIS reflectance and XRF data, so that polyene formations through dehydrochlorination of PVC during alkali attack is one of dominant mechanisms for the performance reduction of the alkali-attacked AMX at 40 °C. These results are promising to be useful for the analysis of ion exchange membranes (IEMs) used in real commercial processes on-site in future.

## 1. Introduction

Electrodialysis (ED) using ion exchange membranes (IEMs) have been widely used as a low-cost efficient water treatment process in various fields such as foods, beverages, chemicals, and so on where salt enrichment and/or desalination are required [1,2,3,4]. More recently, the ED processes are also used in more specialized environmental applications such as lithium recovery from salt lakes [5], desalination for water recovery in shale gas production [6], and carbon dioxide recovery from acid gas in power plants [7,8].

IEMs are mainly classified as two types: hydrocarbon-type (styrene-divinyl-benzene copolymer type) and the others (e.g., ethylene glycol dimethacrylate, sulfoethyl methacrylate, and so on). Because the hydrocarbon-type IEMs are generally more stable in ordinary concentrations of acid and alkali solutions than the other types [9], hydrocarbon-type IEMs are widely applied in the above applications as commercial IEMs. Here, we focused on alkaline attack of hydrocarbon-type IEMs, and eliminated that of perfluoro polymer-type IEMs, which are often used for electrolysis (not water treatment processing). In addition, depending on their structure, IEMs are also classified into homogeneous and heterogeneous ones [9]. The homogeneous IEMs are generally prepared by a paste method and subsequent introducing of an ion-exchange moiety directly into the structure of the constitutive polymer [9]. In the former pasting procedure, a fine mixed solution including monomers (styrene, chloromethyl-styrene divinyl-benzene), initiator, and binder is casted on a polyvinyl chloride (PVC)-based fabric as a backing, and then heated to prepare a precursor membrane film for subsequent introduction of the charged groups, such as sulfonation or amination [4]. Because of this preparation procedure, the homogeneous IEMs have a relatively even distribution of the charged groups over the entire membrane matrix, and it enables the preparation of the IEMs with a thinner thickness, resulting in a higher electrical performance, such as lower membrane resistance [10]. In commercial IEMs, Selemion^®^ AMV (AGC Engineering Co. Ltd., Chiba, Japan) and Neosepta^®^ AMX (Astom Corp., Tokyo, Japan) are regarded as homogeneous ones. Especially regarding the AMX prepared by the paste method; PVC is included in the backing and binder.

In ED applications, stable, reliable, and long lifetime operation is always required for ensuring high production quality and low operational cost. However, ED processes often require some maintenance to recover the system from certain problems that eventually cause performance reduction. Especially, fouling of the anion exchange membrane (AEM) is one of the serious problems that increases the operational cost and shortens the lifetime of the AEM, owing to an unfavorable increase in the electric resistance of the AEM [11]. Commercially, cleaning-in-place (CIP) (i.e., cleaning the inside of an ED stack without disassembly) with chemical agents such as alkali solutions is widely used to recover the ED performance reduced by the membrane fouling of the AEMs. However, simultaneously, it often leads to an unfavorable performance reduction of the AEMs, resulting in shorter lifetime of the AEMs even if the ED performance is completely recovered. On the other hand, the fouling within the spacer between the IEMs (spacer clogging) is also one of the critical problems that impedes the flow pathway, resulting in higher operational costs due to the increase of pumping energy. Furthermore, the spacer clogging also reduces the sufficient turbulence within the flow and eventually causes unfavorable water splitting (H^+^ and OH^−^ generation) because of unfavorable partial shortage of the ions supplied, resulting in shorter lifetime of the AEMs. Also, because the generated OH^−^ preferably immersed into the AEMs due to electrical partitioning and then damages occur [12]. Therefore, in any cases, alkali tolerance of AEM is one of the important issues to achieve reliable and long lifetime in ED operation.

Regarding the alkali attack on the AEMs, some mechanisms of are suggested: (1) Degradation of the anion exchange group (quaternary ammonium group) in unordinary concentration of alkali solution [13]. In this case, AEM performance decreases due to loss of the charged group for ion transportation. (2) Dehydrochlorination of PVC within the backing and binder. In this case, the quaternary ammonium group act as a catalyst for dehydrochlorination of PVC, polyene is subsequently synthesized from PVC, and then, electric performance of the AEMs eventually decrease [14]. However, a relationship between the alkali attack mechanism and the resulting membrane performance has not been systematically investigated yet.

**Figure 1:**
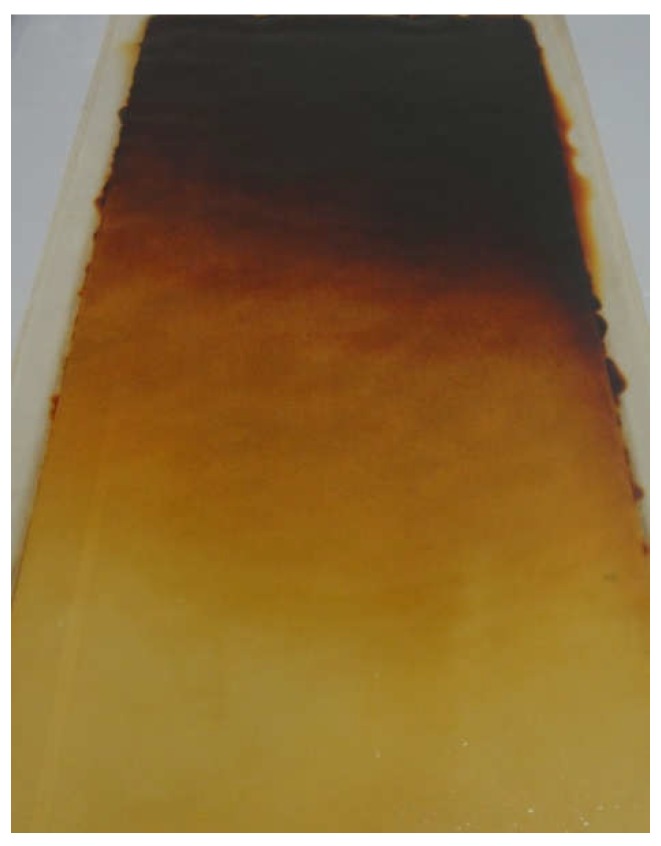
Neosepta^®^ AMX used in electrodialysis (ED) desalination of leachate for 8 years with appropriate cleaning-in-place (CIP) treatments.

For the maintenance, the ED stack is sometimes disassembled when the above problems are quite severe and not solved by CIP. Disassembling enables the direct removal of the foulants on the IEMs and spacers. Meanwhile, color change of the IEMs from transparent to some kinds of color (yellow, orange, red, and dark red) are also often observed as shown in Figure 1 (Neosepta^®^ AMX used in ED desalination of leachate for 8 years). This color change is generally irreversible, and workers on site instinctively would notice that immoderate color change may cause the performance reduction of IEMs. Thus far, in the case of AMX, it was suggested that the color change of AMX was caused by dehydrochlorination of PVC [15,16]. Therefore, dehydrochlorination of PVC in backing and binder and subsequent polyene formation may lead the AMX to mechanically weaken, and the electrical performance of the AMX would eventually reduce. Although workers on site would notice that there is a relationship between the color change and performance reduction of AMX, a destructive sampling (making a specimen for subsequent respective electrochemical analysis) is unavoidable to confirm a final decision whether the colored AMX can be continuously used or not. Therefore, the membrane will be disposed of due to the destructive sampling, even if the electric performance of the colored AMX is enough for the ED process. Therefore, nondestructive and quick diagnosis-in-place (DIP) are required to propose an adequate decision for the colored and potentially deteriorated AMX. However, systematic research on the relationship between color change of AMX and both electrical and mechanical performance has not been fully demonstrated yet.

In our previous study [15], we firstly investigated the relationship between color change of the AMX and electrical performance after alkali attack by means of destructive analysis. The alkali-attacked AMXs with a wide variety of colors were prepared by immersion of the AMX into the alkali solutions under various conditions. After that, the electrical performance of the colored AMX, such as membrane resistance, proton rejection, amount of fixed-charge sites, water content, and Young’s modulus were systematically analyzed. Degree of polyene formation was indirectly quantified by VIS absorbance, and a clear linear relationship was successfully found between VIS absorbance and respective membrane performance, indicating the degree of polyene formation caused the performance reduction of the AMX. However, a nondestructive analytical method for the quick DIP has not been confirmed yet. Therefore, in this study, to develop the nondestructive analysis for the quick DIP, we have further investigated the relationship by means of relative total VIS reflectance and handheld X-ray fluorescence (XRF) analyses.

## 2. Experimental

### 2.1. Membranes and Chemicals

Commercially available homogeneous AEM, Neosepta^®^ AMX (from Astom Corp., Tokyo, Japan), was used in this study. As described above, PVC is included within the backing and binder of the AMX. The PVC within the binder and ion exchange resin are homogeneously intertwined within a nano-size scale [15]. Unless otherwise specified, the AMX with its counter ion of Cl^−^ was used for the alkali immersion test and subsequent optical analyses by immersing the membrane into 0.5 M NaCl solution for at least 24 h.

Hydrochloric acid (HCl), sodium chloride (NaCl), sodium hydroxide (NaOH), and sodium nitrate (NaNO_3_) obtained from Wako Pure Chemical Industries (Osaka, Japan) were used as necessary for the membrane sample preparation. De-ionized water with the electric conductivity of approximately 5 μS/cm was used to prepare all sample solutions. Unless otherwise specified, all the reagents were of analytical grade and used without further purification.

### 2.2. Alkali Immersion Test

The alkali immersion test was conducted as described in our previous report [15]. Briefly, AMXs with the counter ion of Cl^−^ were immersed in 0 M (water), 0.01 M, 0.1 M, and 1 M NaOH at 40, 60, and 80 °C (totally, 12 conditions). The immersed sample was taken at the immersion time of 1, 3, 5, 24, 48, and 168 h. After sampling, the AMX was immersed in 0.5 M NaCl for 24 h, washed with a sufficient amount of de-ionized water, and carefully wiped to remove the surplus water on the membrane surfaces for the following measurements.

### 2.3. Membrane Characteristics and Performance after Alkali Immersion

Electrical and mechanical performance data such as membrane resistance, proton rejection, amount of fixed-charge sites, and Young’s modulus of the AMXs after the alkali immersion tests were provided from our previous literature [15]. The measurements for the electrical and mechanical performance of the membrane were performed according to the literature [13,16]. To compare the membrane performance of before and after the alkali immersion test, we calculated the normalized performance ratio (PR) as follows:

(1) $$PR=\frac{P_{after}}{P_{before}}$$

where *P_after_* and *P_before_* are performance (e.g., membrane resistance, proton rejection, amount of fixed-charge sites, and Young’s modulus) after and before alkali immersion test, respectively.

### 2.4. Optical Analyses for Alkali Immersed AMXs

#### 2.4.1. VIS Reflectance Measurement

Relative total VIS reflectance, including both diffuse and specular ones, at 600 nm of the alkali-immersed AMXs was measured using a UV-VIS spectrometer (UV-2600, Shimadzu Corporation, Kyoto, Japan) equipped with an integrating sphere unit (ISR-2600, Shimadzu Corporation, Kyoto, Japan). The relative total VIS reflectance was measured with the incident angle of 8°. A powder sampling holder (P/N206-21865-91, Shimadzu Corporation, Kyoto, Japan) filled with barium sulfate (BaSO_4_) powder and a standard white plate (total reflectance = 100%) was used as a reference reflectance sample. Before measurement, membranes with the counter ion of Cl^−^ were vacuum dried for at least 4 h.

#### 2.4.2. XRF Measurement

Element composition within the alkali-immersed AMX was analyzed using a handheld X-ray fluorescence device (Vanta Handheld XRF series, Olympus Corporation, Tokyo, Japan). The handheld XRF apparatus recently enables metal or nonmetal identification in a minute, and it may allow compliance testing on-site as a reliable DIP A fluorescent X-ray emission (2.6 keV for Cl Ka1) was measured and used for analysis for the dehydrochlorination within the AMX. To discuss the degree of dehydrochlorination, we calculated the normalized ratio by comparing the Cl amounts (intensity) before and after alkali immersion tests. Since the counter ions of the AMX were fixed with Cl^−^, the quantitative amount of Cl obtained from XRF analysis was originated from not only Cl within the PVC in the backing and binder, but also the counter ions (equal to the amount of fixed-charge sites of the AMX). Before measurement, the membranes with the counter ion of Cl^−^ were also vacuum dried for at least 4 h.

## 3. Results and Discussion

### 3.1. Color Change of the Alkali Attacked AMX

**Figure 2:**
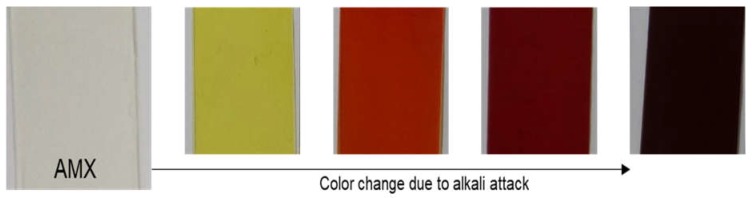
A color change trend of commercial AMX during the alkali immersion test.

Figure 2 shows a color change trend of the AMXs during the alkali immersion test. The color of the AMXs changed gradually from transparent to yellow, orange, red, and dark red with increasing immersion time period, and finally became dark violet. As the duration, NaOH concentration, and temperature increased, the color change of AMXs became faster. Furthermore, this color change was irreversible and entirely occurred over all of the membranes [15]. Therefore, this uniform and gradual color change within the entire membrane due to the polyene formation may allow a quantitative analysis by using an optical device, such as VIS reflectance and XRF analyses. For the VIS reflection measurement, since the wavelength of the color (visible light reflection) gradually increased in the order of yellow (570–590 nm), orange (590–620 nm), red (620–750 nm), and dark red (>750 nm), VIS reflection at 600 nm was adopted as the suitable condition according to previous literature [15].

### 3.2. VIS Reflectance Results

#### 3.2.1. Time Course under Different Immersion Conditions

**Figure 3:**
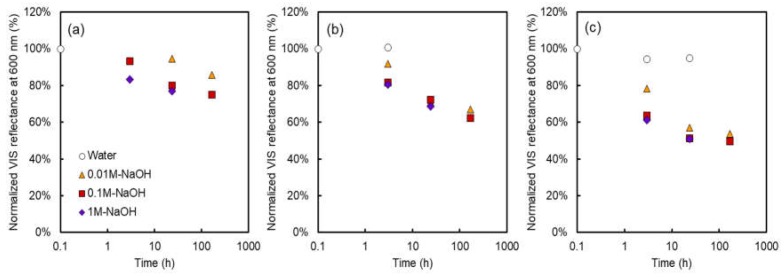
Normalized relative total visible (VIS) reflectance as a function of the time after immersion into NaOH solution. The temperature of the alkali immersion test: (**a**) 40 °C, (**b**) 60 °C, (**c**) 80 °C.

Figure 3 shows the normalized relative total VIS reflectance at 600 nm using the alkali-immersed AMXs under various immersing times, NaOH concentrations, and temperatures. In the case of the test pieces immersed in water, almost no change was observed in the VIS reflectance, even at 80 °C (highest temperature condition in this study, which is much higher than the 40 °C recommended maximum temperature from the membrane manufacturing company [15]). When the normalized VIS reflectance was 100%, the value of the relative total VIS reflectance was 8.8%. On the other hand, in the case of the test pieces immersed in NaOH solutions, the normalized VIS reflectance apparently decreased, depending largely on the temperature of the immersed solution. In the presence of NaOH in the immersed solution, the normalized VIS reflectance decreased with an increase in the immersing time, temperature of the immersed solution, and NaOH concentration.

#### 3.2.2. Correlation to the Performance of the Alkali-Attacked AMXs

**Figure 4:**
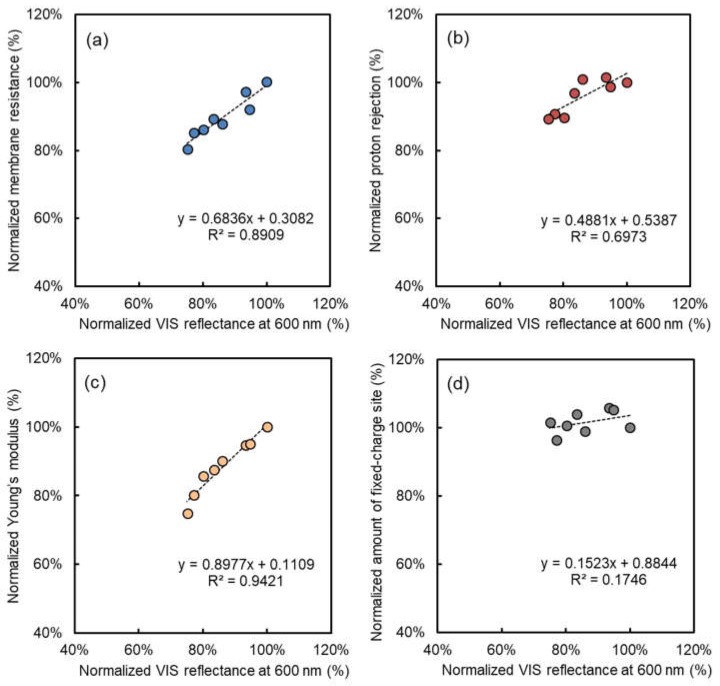
Relationships between electrical and mechanical properties and normalized relative total VIS reflectance at 600 nm of AMX after immersion tests into NaOH solution at 40 °C: normalized (**a**) electrical resistance, (**b**) proton rejection, (**c**) Young’s modulus, and (**d**) amount of fixed-charge sites.

**Figure A1:**
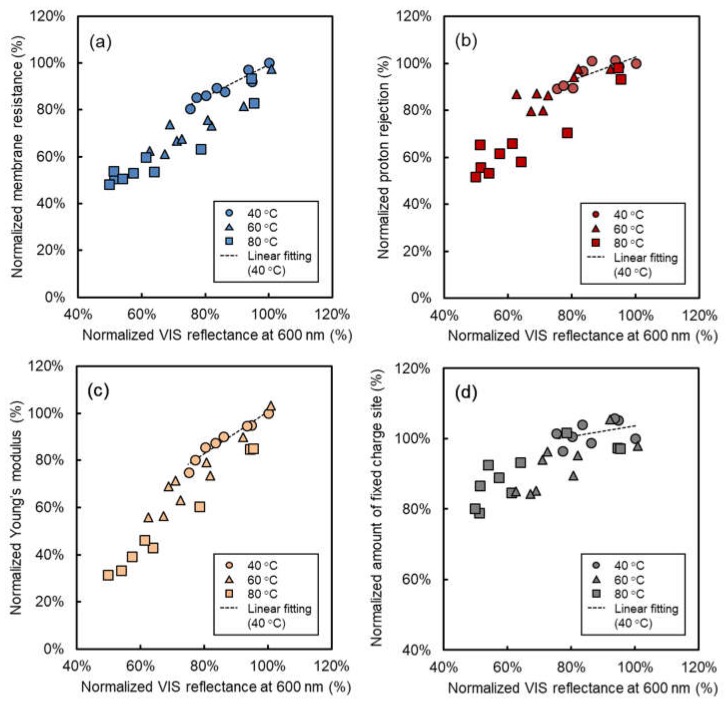
Correlation between normalized electrical and mechanical performance and normalized VIS reflectance at 600 nm after NaOH immersion at 40 °C, 60 °C, and 80 °C: normalized (**a**) electrical resistance, (**b**) proton rejection, (**c**) Young’s modulus, and (**d**) amount of fixed-charge sites.

Figure 4 shows the relationship between normalized relative total VIS reflectance results and the electrical and mechanical performance data (membrane resistance, proton rejection, amount of fixed-charge sites and Young’s modulus) of the alkali-immersed AMXs at 40 °C. The same relationships, including the 60 °C and 80 °C cases, are also shown in Figure A1. The membrane resistance, proton rejection, and Young’s modulus of the alkali-attacked AMXs definitely decreased with the increasing NaOH concentration, immersion time, and temperature. On the other hand, the amount of fixed-charge sites was almost stable in the 40 °C case, and clearly decreased in the 60 °C and 80 °C cases. Because the amount of fixed-charge sites is the total amount of the charged group within the membrane obtained by a titration method [14], a decrease in the amount of fixed-charge sites must be due to deterioration of the charged group. In the case of the immersion at 40 °C, the change in the amount of fixed-charge sites was not severe and can be negligible during alkali immersion. Therefore, in this case, it was suggested that polyene formation due to dehydrochlorination of PVC would be dominant for the resulting electrical and mechanical performance of the alkali-attacked AMXs, whereas deterioration of the charged group did not occur. On the other hand, the decrease of the amount of fixed-charge sites was not negligible during alkali immersion at 60 °C and 80 °C as shown in Figure A1.

In Figure 4, interestingly, clear linear correlations with acceptable deviations were obtained on the membrane resistance, proton rejection, and Young’s modulus of the alkali-attacked AMXs at 40 °C. Therefore, this result clearly indicated that despite different immersed conditions (NaOH concentration and times), the degradation mechanism and subsequent reduction in the electrical and mechanical performance of the membrane would be the same as in the 40 °C case. In addition, because the change in the VIS reflectance at 600 nm was originated from the formation of polyene [15], there is a certain possibility that the polyene formation leads the performance reduction of the alkali-attacked AMX. Up to now, we have proposed an acceptable mechanism for the performance reduction due to alkali attack, especially at 40 °C [15]: (1) formation of polyene triggers the reduction of Young’s modulus (the mechanical property of the membrane becomes weak and brittle [13]); (2) reduction of Young’s modulus subsequently leads to an increase of water content; and then (3) the increase of water content eventually causes a reduction of the electrical performance. In AMX, the PVC backing and fabric actually have a key role to hold the fixed ionic charge with high ionic concentration against the swelling due to osmotic pressure. Therefore, the loss of mechanical strength of the PVC backing and fabric due to polyene formation will lead the swelling of the membrane, and the increase of the swelling ratio subsequently will cause the performance reduction.

When the immersion temperature is higher than 40 °C, such as 60 °C and 80 °C (higher than the commercially recommended temperature), in addition to the above mechanisms, a release of residual strain within the AMX by a heat-induced annealing (annealing effect) would further occur, eventually resulting in an additional reduction of Young’s modulus and subsequent electrical performance [15]. Therefore, it is worth noting that the alkali attack mechanism on the resulting performance at 40 °C is different from those at 60 °C and 80 °C, and therefore, different correlations were observed in the case of 60 °C and 80 °C compared with the 40 °C case, as shown in Figure A1. In the view point of commercial applications, the correlation between VIS reflectance and resulting performance at 40 °C is most important for quick DIP because the recommended available temperature for the AMX is less than 40 °C. In the case of immersion temperature at 40 °C, its deviation in the respective linear correlation was relatively low, and the range of the measured normalized reflectance was also relatively wide (75–100%). Therefore, these results clearly showed a good possibility that usage of a portable VIS spectrophotometer is promising to be useful as a quick DIP in the future. To develop the quick DIP by VIS reflection approach, the portable VIS analysis for the IEMs used in commercial processes on-site for several years with appropriate CIP treatments should be also conducted in future.

### 3.3. Handheld XRF Results

#### 3.3.1. Time Course under Different Immersion Conditions

**Figure 5:**
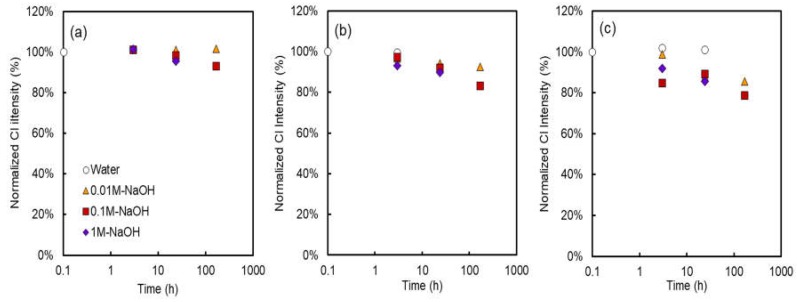
Normalized chloride intensity as a function of the time, concentration, and temperature of the alkali immersion test: (**a**) 40 °C, (**b**) 60 °C, (**c**) 80 °C.

Figure 5 shows the normalized Cl intensity, (after immersion test/original) of the alkali-immersed AMXs under various immersing times, NaOH concentrations, and temperatures. As expected, in the case of the test pieces immersed in water, almost no reduction in the Cl intensity was observed even at 80 °C, similar to the VIS reflectance results. Therefore, this result also supported that both polyene formation and elimination of the ion exchange site did not occur in the absence of NaOH. On the other hand, in the case of the test pieces immersed in NaOH solution, the Cl intensity clearly decreased, that is, dehydrochlorination of PVC (polyene formation) and/or elimination of the charged group occurred. At least, dehydrochlorination of PVC and subsequent polyene formation has undoubtedly occurred through the following reaction [15,17] because the VIS reflectance was also decreased:

-CHCl-CH_2_- → -CH = CH- + HCl (2)

Since the dehydrochlorination changes the physicochemical properties of PVC within the backing and binder, the resulting electrical and mechanical properties of the AMX will change depending on the dehydrochlorination degree. On the other hand, since the Cl intensity includes Cl of the counter ions, the elimination of the charged group due to Hoffman elimination mechanisms [15] possibly occurred. However, since our preliminary estimation indicated the amount of Cl originated from PVC was about nine times higher than that from the counter ion within the AMX, the small amount of elimination of the charged group may be difficult to quantitatively detect by means of XRF in the presence of dehydrochlorination of PVC.

#### 3.3.2. Correlation to the Performance of the Alkali Attacked AMXs

**Figure 6:**
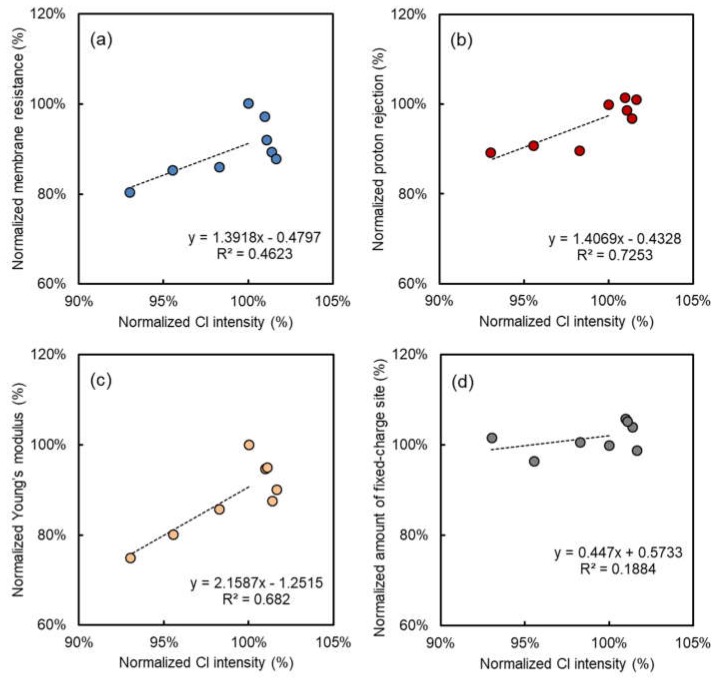
Correlation between electrical and mechanical properties and normalized chloride intensity after NaOH immersion at 40 °C: normalized (**a**) electrical resistance, (**b**) proton rejection, (**c**) Young’s modulus, and (**d**) amount of fixed-charge sites.

**Figure A2:**
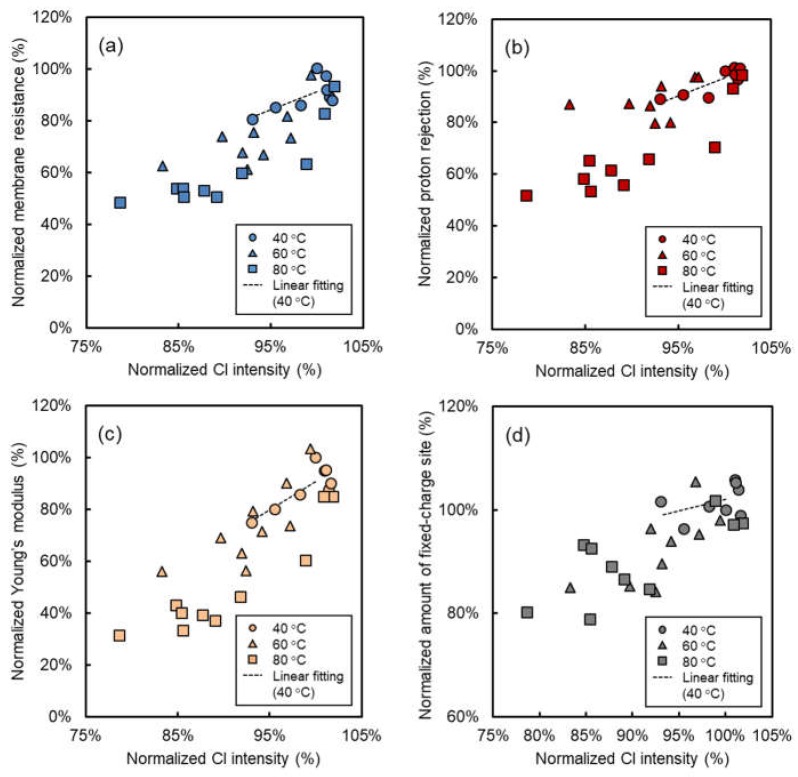
Correlation between normalized electrical and mechanical performance and normalized Cl intensity after NaOH immersion at 40 °C, 60 °C and 80 °C: normalized (**a**) electrical resistance, (**b**) proton rejection, (**c**) Young’s modulus, and (**d**) amount of fixed-charge sites.

Figure 6 shows the relationship between handheld XRF results and all membrane performance data at 40 °C, such as membrane resistance, proton rejection, amount of fixed-charge sites, and Young’s modulus of the alkali-immersed AMXs under various immersing conditions provided from our previous literature [15]. The same relationships (normalized Cl intensity vs. all membrane performance data) at 60 °C and 80 °C are also shown in Figure A2. Regarding the XRF results, linear correlations were also obtained as well as the VIS reflectance case. Therefore, the results also supported that the handheld XRF analysis can mainly detect the dehydrochlorination due to the polyene formation in the 40 °C case because the amount of fixed-charge sites was almost stable. On the other hand, in the 60 °C and 80 °C cases, the amount of fixed-charge sites apparently decreased after alkali immersion as shown in Figure A2, and therefore, the XRF would detect both the dehydrochlorination of PVC and the decrease of the counter Cl ion, although the total amount of Cl originated from PVC was about nine times higher than that from the counter ion. To confirm this, we also conducted the XRF analysis using alkali-attacked AMXs with the counter ion of NO_3_^−^. Consequently, the Cl intensity of AMXs with NO_3_^−^ was always lower than those of Cl (data not shown). Therefore, it was confirmed that the decrease of the Cl intensity in the handheld XRF analysis also included the decrease of the counter ion of Cl within the alkali-attacked AMXs.

In Figure 6, its deviation in the linear correlation was relatively higher than those in VIS reflectance cases, and also the variation range of the obtained results was relatively narrow (93–103%). Therefore, it indicated that the correlation of electrical and mechanical performance of the alkali-attacked AMX with the handheld XRF data is less reliable than those with VIS reflectance data. Nevertheless, it is worth mentioning that the discriminable and qualitative linear results can be obtained by means of a handheld XRF even in the presence of the counter ion of Cl, that is most reasonable as a model case for commercial use on-site. Furthermore, a handheld XRF also has potential to provide additional useful information, such as composition of scaling and partitioning specific counter ions on site at the same time because the respective information can be easily identified from the characteristic X-ray signals.

### 3.4. Comparison between VIS Reflectance and Handheld XRF Results

**Figure 7:**
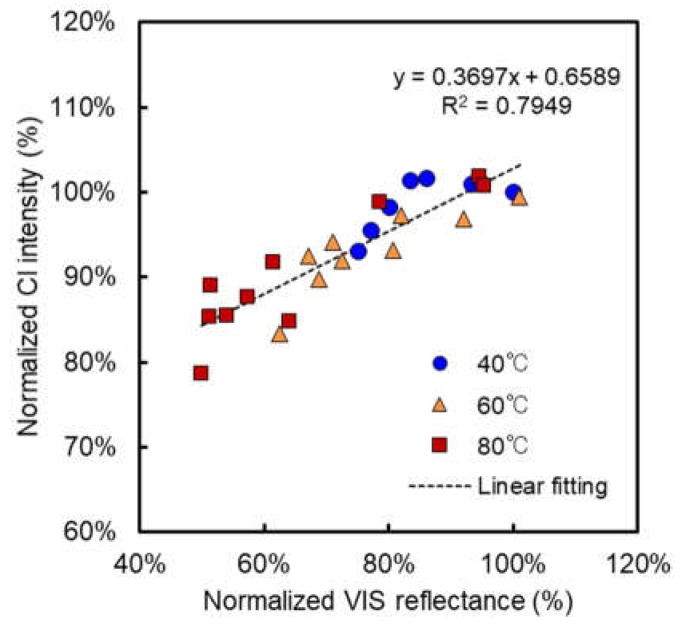
A relationship between normalized relative total VIS reflectance and normalized Cl intensity for all immersion conditions.

Figure 7 shows a correlation between normalized relative total VIS reflectance at 600 nm and normalized Cl intensity for the alkali-attacked AMXs in all immersion conditions at 40, 60, and 80 °C. Here, when the polyene formation is dominant during the alkali degradation of AMX, a clear linear relationship must be obtained because of the 1-1 reaction between the dehydrochlorination of PVC and polyene formation as shown in Equation (2), whereas the linear relation will be obscure when the elimination of the charged group is dominant. As expected, a clear linear relationship was obtained despite different immersion conditions, indicating that the color change due to the polyene formation and dehydrochlorination has a clear interrelation during alkali attack on AMXs. In addition, a single line can be drawn on all the data even at different temperatures with the acceptable degree of the slope of the linear line (>0.25 (=25%)). Therefore, this result indicated that both VIS reflectance and handheld XRF analyses have a good possibility as a useful tool to predict the resulting membrane performance.

Table 1 summarized the comparison of the analyses for alkali-attacked IEMs in order to predict the resulting membrane performance. Up to now, our results showed that VIS spectroscopy and XRF analyses are useful and enable the prediction of the resulting membrane performance of the alkali-attacked AMX, including PVC. Since PVC backing and/or binder are also used in the other commercial IEMs like AMX, VIS reflectance and XRF approaches have a certain versatility and will be also applied for the performance prediction after alkali attack of the commercial IEMs including PVC in the future as quick and nondestructive reliable DIP.

## 4. Conclusions

In this study, we conducted VIS reflectance and handheld XRF analyses in order to compare the electrical and mechanical performance of AMXs after immersion into NaOH solution under various conditions. Because the AMX includes PVC in both backing and binder, relative total VIS reflectance (after immersion/original) provided a degree of polyene formation due to dehydrochlorination of PVC, whereas XRF analysis provided a changing amount of Cl within the AMX. Interestingly, there was a clear linear correlation between these optical data and resulting performance, such as membrane resistance, proton rejection, amount of fixed-charge sites, and Young’s modulus of the alkali-attacked AMXs, especially at 40 °C. The linear relation with VIS reflectance showed lower deviation and a wider changing range than those with XRF, whereas discriminable and qualitative linear results were obtained even by means of handheld XRF. In addition, a clear linear relationship between normalized VIS reflectance and handheld XRF data was found, and it clearly indicated the polyene formation due to dehydrochlorination of PVC is dominant during an alkali attack on AMXs. Moreover, it was found that despite a linear correlation between VIS and XRF results in all temperatures (40, 60, and 80 °C), mechanisms of performance reduction at the respective temperatures were different. The obtained data also allowed the proposal of a reasonable mechanism to understand the reduction of the resulting performance of the alkali-attacked AMXs. This study will be helpful for the analysis of IEMs used in commercial processes on-site for several years with appropriate CIP treatments in future as a nondestructive quick DIP.

## Figures and Tables

**Table 1:** Comparison of the analyses for alkali-attacked ion exchange membranes (IEMs) to predict the resulting performance. DIP: diagnosis-in-place; PVC: polyvinyl chloride.

Methods		Used IEMs	Measurable	Possibility as DIP	Reference
UV spectroscopy	Absorbance	Neosepta^®^ AMX	Amount of polyene formation due to dehydrochlorination from PVC	No	[15]
	Transmittance	-		No	-
	Reflectance (relative total)	Neosepta^®^ AMX		Yes	**In this study**
X-ray fluorescence	Cl intensity (2.6 keV, Ka1)	Neosepta^®^ AMX	Total Cl amount within the membrane	Yes	**In this study**

## Appendix A

### Appendix A1. Relationship between Normalized VIS Reflectance and Resulting Performance of the AMX after Alkali Attacking

Figure A1 shows the relationship between membrane performance and normalized VIS reflectance at 600 nm after NaOH immersion at 40, 60, and 80 °C. A linear fitting line for the 40 °C case is also drawn in this figure. The performance reduction became more severe with increasing immersion temperature, and the slopes for the 40, 60, and 80 °C cases were different. This result indicates that the presence of an annealing effect, which eventually leads to the additional performance reduction in addition to that started from dehydrochlorination of PVC.

### Appendix A2. Relationship between Normalized Cl Intensity and Resulting Performance of the AMX after Alkali Attacking

Figure A2 shows the relationship between normalized Cl intensity (after/before NaOH immersion) and all membrane performance data after NaOH immersion at 40, 60, and 80 °C. A linear fitting line for the 40 °C case is also drawn in this figure. Similar to the UV reflectance case, Cl dependences on the resulting performance in 40, 60, and 80 °C cases were also different. This result indicates that the presence of deterioration of fixed ion-exchange sites, which also eventually leads to the additional performance reduction in addition to that started from dehydrochlorination of PVC.

## References

[1] Strathmann H. (2004). Ion-Exchange Membrane Separation Processes.

[2] Strathmann H. (2010). Electrodialysis, a mature technology with a multitude of new applications. Desalination.

[3] Xu T. (2005). Ion exchange membranes: State of their development and perspective. J. Membr. Sci..

[4] Tanaka Y. (2015). Ion Exchange Membranes Fundamentals and Applications.

[5] Nie X.-Y., Sun S.-Y., Song X., Yu J.-G. (2017). Further investigation into lithium recovery from salt lake brines with different feed characteristics by electrodialysis. J. Membr. Sci..

[6] Peraki M., Ghazanfari E., Pinder G.F., Harrington T.L. (2016). Electrodialysis: An application for the environmental protection in shale-gas extraction. Sep. Purif. Technol..

[7] Taniguchi I., Yamada T. (2017). Low energy CO_2_ capture by electrodialysis. Energy Procedia.

[8] Dara S., Lindstrom M., English J., Bonakdarpour A., Wetton B., Wilkinson D.P. (2017). Conversion of saline water and dissolved carbon dioxide into value-added chemicals by electrodialysis. J. CO_2_ Util..

[9] Sata T. (2004). Ion Exchange Membranes: Preparation, Characterization, Modification and Application.

[10] Garcia-Cesquez W., Ghalloussi R., Dammak L., Larchet C., Nikonenko V., Grande D. (2014). Structure and properties of heterogeneous and homogeneous ion-exchange membranes subjected to ageing in sodium hypochlorite. J. Membr. Sci..

[11] Tanaka N., Higa M. (2011). Organic fouling properties of anion-exchange membranes with various electrodialysis conditions. Bull. Soc. Sea Water Sci. Jpn..

[12] Choi J.-H., Moon S.-H. (2003). Structural change of ion-exchange membrane surfaces under high electric fields and its effects on membrane properties. J. Colloid Interface Sci..

[13] Sata T., Tsujimoto M., Yamaguchi T., Matsusaki K. (1996). Change of anion exchange membranes in an aqueous sodium hydroxide solution at high temperature. J. Membr. Sci..

[14] Garcia-Vasquez W., Dammak L., Larchet C., Nikonenko V., Pismenskaya N., Grande D. (2013). Evolution of anion-exchange membrane properties in a full scale electrodialysis stack. J. Membr. Sci..

[15] Doi S., Yasukawa H., Kakihana Y., Higa M. (2019). Alkali attack on anion exchange membranes with PVC backing and binder: Effect on performance and correlation between them. J. Membr. Sci..

[16] Tanaka N., Nagase M., Higa M. (2011). Preparation of aliphatic-hydrocarbon-based anion-exchange membranes and their anti-organic-fouling properties. J. Membr. Sci..

[17] Garcia-Vasquez W., Dammak L., Larchet C., Nikonenko V., Grande D. (2016). Effects of acid-base cleaning procedure on structure and properties of anion-exchange membranes used in Electrodialysis. J. Membr. Sci..

